# The Journal of Botany

**Published:** 1834-04-01

**Authors:** 


*The Journal of Botany, being a Second Series of the Botanical Miscellany.* Part I. (To be continued Quarterly.) By WM. JACKSON HOOKER, Regius Professor of Botany in the University of Glasgow.—London, 1834. 8vo. pp. 96, and eight Plates.

DR. HOOKER's name has become so identified with botanical excellence, that it is almost a sufficient recommendation of this elegant periodical to quote its title-page. The first article is an able account of the genus Floerkea of Willdenow, by Professor Lindley. He thinks that this genus must be placed among the Sanguisorbeæ, or in their neighbourhood. There is an interesting "notice concerning Mr. Drummond's collections, made in the southern and western parts of the United States." Mr. Drummond, it appears, is still in America, and we have little doubt that the researches of this enthusiastic botanist will be rewarded by a rich harvest of discoveries.

Two of the plates are especially beautiful: they are coloured representations of two new ferns, the *Gymnogramma elongata*, and *G. flabellata.*

